# DNA binding of an RNA helicase bacterial transcription terminator

**DOI:** 10.1042/BCJ20240452

**Published:** 2025-01-31

**Authors:** Sriyans Jain, Abhijeet Behera, Ranjan Sen

**Affiliations:** 1 Laboratory of Transcription, Centre for DNA Fingerprinting and Diagnostics, Inner Ring Road, Uppal, Hyderabad, 500039, India; 2 Centre for Doctoral Studies, Manipal Academy of Higher Education, Manipal, Karnataka, India

**Keywords:** ATPase, gel-shift assays, SIM fluorescence microscopy, transcription termination

## Abstract

The bacterial transcription terminator Rho is a hexameric ATP-dependent RNA helicase that dislodges elongating RNA polymerases. It has an N-terminal primary RNA binding site (PBS) on each subunit and a C-terminal secondary RNA binding site at the central channel. Here, we show that Rho also binds to linear longer double-stranded DNAs (dsDNAs) and the circular plasmids nonspecifically using its PBS. However, this interaction could be competed efficiently by single-stranded DNA (ssDNA), dC_34_. Long dsDNA (3.5 kb) at the PBS activates short oligo C RNA-mediated ATPase activity at the secondary binding site (SBS). The pre-bound Rho to this long DNA reduces the rate and efficiency of its transcription termination activities *in vitro*. Elevated concentrations of Rho reduced the *in vivo* transcription level suggesting that Rho might also function as a nonspecific repressor of gene expression under certain conditions. In the mid-log phase culture, Rho molecules were concentrated at the poles and along the membrane. In contrast, the Rho hexamers were observed to be distributed over the bacterial chromosome in the stationary phase likely in a hyper-oligomeric state composed of oligomers of hexamers. We propose that Rho molecules not engaged in the transcription termination process could use the bacterial chromosome as a “resting surface”. This way the “idle” DNA-bound Rho molecules could be kept away from accidentally loading onto the nascent RNA and initiating unwanted transcription termination.

## Introduction

Rho is a homo-hexameric transcription termination factor that dislodges the transcription elongation complex (EC) using its RNA helicase activity [1,2]. Rho binds to the nascent RNA through its N-terminal primary RNA binding sites (PBSs) that is composed of an N-terminal helix bundle (NHB) and five-stranded β-barrel structure (oligonucleotide binding fold, OB-fold) [3–5]. NHB had been proposed to channel the nucleic acids to the OB-fold [5]. The nascent RNA is eventually chaperoned through its central cavity, interacting with the secondary binding sites (SBSs) and activating its ATPase and helicase activities. Utilizing the energy released by ATP hydrolysis, Rho then translocates toward the transcribing RNA polymerase to terminate the transcription [3]. Rho termination has been implicated in various cellular functions, including limiting pervasive transcription, removing R-loops, maintaining genome integrity, and repairing DNA [1,6–12].

Although Rho binding to RNA is the most commonly thought mechanism of Rho termination, the alternate model proposes that Rho initially interacts with the RNAP near the promotor region and later transferred to the emerging RNA [13–16]. Genome-wide ChIP-Seq assays in *Escherichia coli* and *Mycobacterium tuberculosis* showed Rho distribution throughout the genome with the peak signal near promotor regions of the genes overlapping the signal of RNAP [17,18]. *In vitro* experiments showed that Rho is capable of binding to ssDNA at its PBS [19–21]. These data led us to hypothesize that Rho may also interact with double-stranded DNA (dsDNA) influencing the global gene regulation *in vivo*. We also hypothesized that outside the EC, free Rho hexamer could bind to the bacterial chromosome. To test these hypotheses, it would be required to undertake a thorough Rho–DNA interaction study both *in vitro* and *in vivo*.

Here, we show that Rho can bind to linear long dsDNAs and the circular plasmids nonspecifically utilizing its N-terminal PBS. However, this interaction could be competed efficiently by ssDNA, dC_34_. The presence of long dsDNA at the PBS could activate short oligo rC RNA-mediated ATPase activity at the SBS. A partial reduction in the rate and efficiency of Rho-dependent transcription termination was observed when Rho was bound to the linear DNA before initiating its termination function. This Rho–DNA interaction also led to the reduction in the *lacZ* gene expression *in vivo*. Rho molecules in the mid-log phase culture were observed to be concentrated at the poles and along the membrane, and they were observed to be distributed over the bacterial chromosome in the stationary phase.

## Results

### *In vitro* dsDNA binding function of Rho

**Figure 1:**
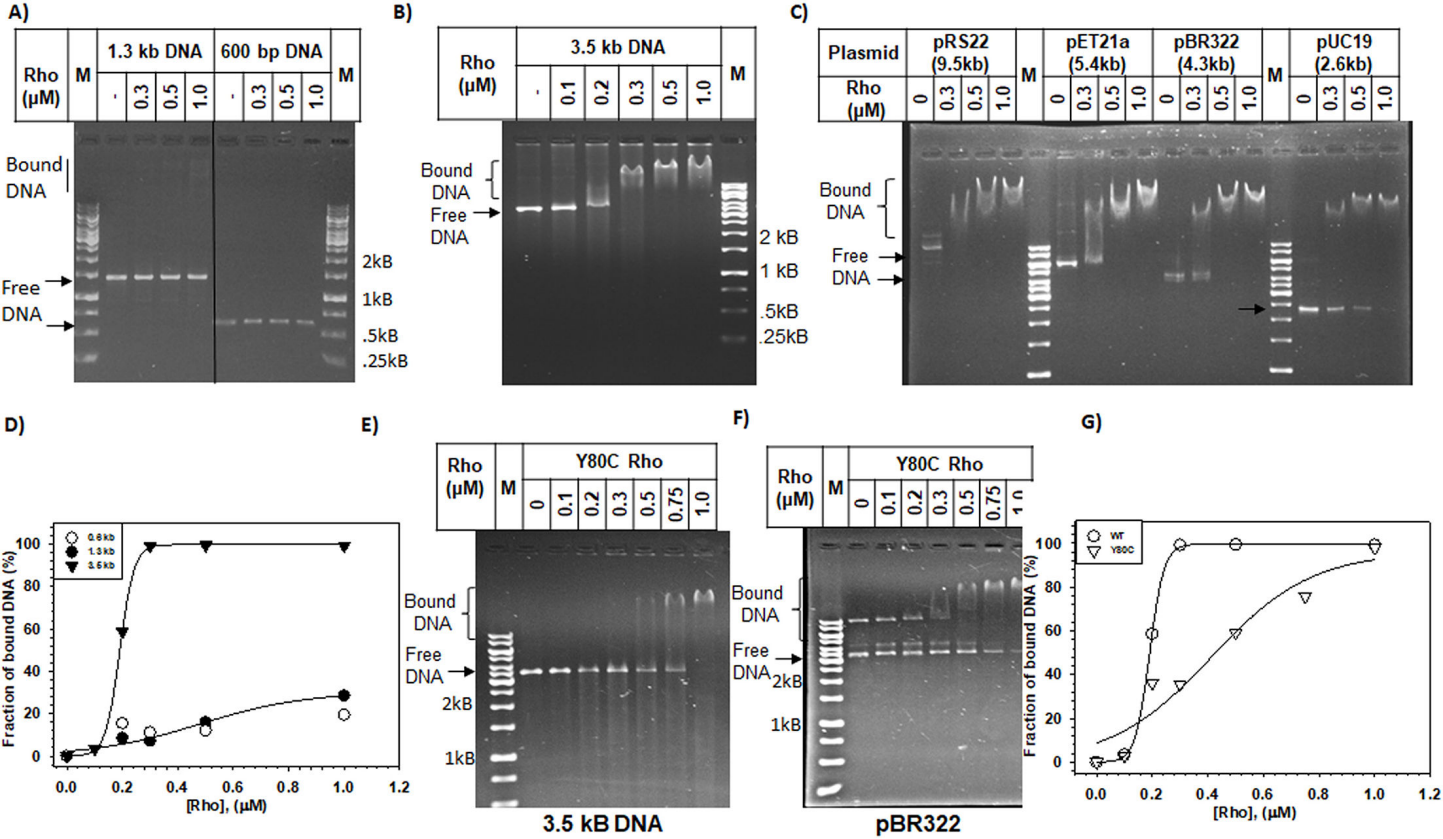
Gel shift assay for Rho binding with dsDNAs of different lengths. (**A**) DNAs of length 600 bp and 1.3 kb showed little or no shift in the band position on the addition of Rho indicating Rho does not bind with these DNAs. Longer DNA of 3.5 kb (**B**) and different circular plasmids (**C**) showed band shift with the increasing concentrations of Rho indicating the formation of the Rho-–DNA complex. (**D**) Binding isotherms of Rho–-dsDNA interactions for the DNA fragments are indicated. Gel- shift assays of the interaction of Rho PBS mutant, Y80C, with (**E**) 3.5 kb linear dsDNA and (**F**) with the plasmid pBR322 DNA. (**G**) Binding isotherm of the WT and Y80C Rho interactions with the 3.5 kB dsDNA.

To demonstrate direct interactions of Rho with different double-stranded (ds) linear DNA molecules of varied sequences and lengths (Figure 1A, B, and D, Supplementary Figure S1A and S1B)*,* we chose 600 bp, 1.3 kb, 1.5 kb, 2.0 kb, and 3.5 kb linear dsDNA fragments. We added increasing concentrations of Rho to a fixed concentration of these DNA fragments. Rho showed no (Figure 1A and D) or very weak binding (Supplementary Figure S1A, C and Table S1) to the smaller dsDNAs of<1.5 kb (Figure 1A and Supplementary Figure S1A, C and Table S1A) but interacted efficiently with the longer DNA fragments of 2 kb and 3.5 kb. (Figure 1B and D and Supplementary Figure S1B, C and Table S1A). The band position of the 3.5 kb DNA–Rho complex shifted gradually to the positions corresponding to higher molecular weight with increasing concentrations of Rho. It reached saturation at 1 μM of Rho hexamer manifested as super-shifted bands, suggesting multiple Rho molecules binding to this longer DNA. The minor variations in the weak binding affinity of Rho observed between the two 0.6 kB DNA fragments shown in Figure 1A and Supplementary Figure S1A were because in one case it was PCR-amplified from pRS22 using the primer pairs RS58/RK23B (Figure 1A; Supplementary Table S2) and in another case, it was amplified from the gDNA of the MG1655 strain using the primer pair RS300/RS1744 (Supplementary Figure S1A and Table S2).

We also checked the interaction of Rho with plasmids (circular DNA) of different sizes. Similar to the linear DNA, plasmid DNAs also showed mobility shifts with increasing concentrations of Rho suggesting their efficient binding (Figure 1C). Our plasmid preparations predominantly consist of supercoiled plasmids; hence, we observed Rho’s interaction primarily with this form. These results suggested that multiple Rho molecules are capable of binding to different plasmids and long linear DNA molecules in a nonspecific manner. The absence of or very weak binding to 0.6 kB or 1.3 kB DNA fragments and observation of stable binding to 1.5 kB DNA and higher fragments could be because of the requirement of cooperative binding among the multiple Rho hexamers. These cooperative interactions of Rho molecules on the surface of the long DNA could lead to hyper-oligomerization of each Rho hexamers, which might have appeared as super-shifted bands on the gels and as sigmoidal binding curves. The longer DNA fragments were required to achieve a stable cooperative DNA–Rho hexamer interaction. Since there is a minor difference in the binding affinities between the two 0.6 kB fragments with different sequences, certain DNA sequences might also contribute to the stability of Rho–dsDNA binding. Although these binding data do not eliminate the possibility that the single Rho hexamer binds to multiple DNA molecules, it is not likely to occur due to steric hindrances from the presence of more than one DNA molecule bound to the closely spaced PBSs in each subunit of a Rho hexamer.

According to the crystal structures of the nucleic acid-bound Rho hexamers, the PBS accommodates both ssDNA and RNA oligonucleotides, whereas its central channel containing the secondary nucleic acid binding sites (SBS) accommodates only the ssDNA [19,22,23]. We hypothesized that the PBS would be able to accommodate a dsDNA, and Rho mutants in the PBS domain having weaker affinity for the nucleic acids would also have reduced affinity for the dsDNA. We used a Rho PBS mutant, Y80C [20], that has weaker affinities for the ssDNA/ssRNA at the PBS. Rho–NTD–nucleic acid complex structure showed that the Y80 residue makes direct contact with the bound nucleic acid [23]. Gel shift assays with 3.5 kB linear DNA and the plasmid DNA (Figure 1E–G) revealed that this Rho mutant has ~7-fold weaker binding to these DNA molecules (Supplementary Table S1B). This result suggests that dsDNA prefers to bind the Rho-NTD having the PBS.

It is possible that at 37°C, the long linear dsDNA or the plasmid DNA tends to form single-stranded regions due to breathing and fraying at the DNA ends, which could be the targets of Rho in the above experiments. Therefore, we repeated the binding assays of Rho with the 3.5 kB linear and the pBR322 plasmid DNA at 4°C where the DNA breathing and end fraying is likely to be minimal. We observed that the Rho–DNA complex formations occurred with a twofold higher affinity at 4°C compared with that was observed at 37°C (Supplementary Figure S1D, E and Table S1A). The tighter binding at low temperatures where DNA is predominantly in the ds form further supports our conclusion that Rho is preferably bound to the ds regions of the long DNA molecules.

**Figure 2:**
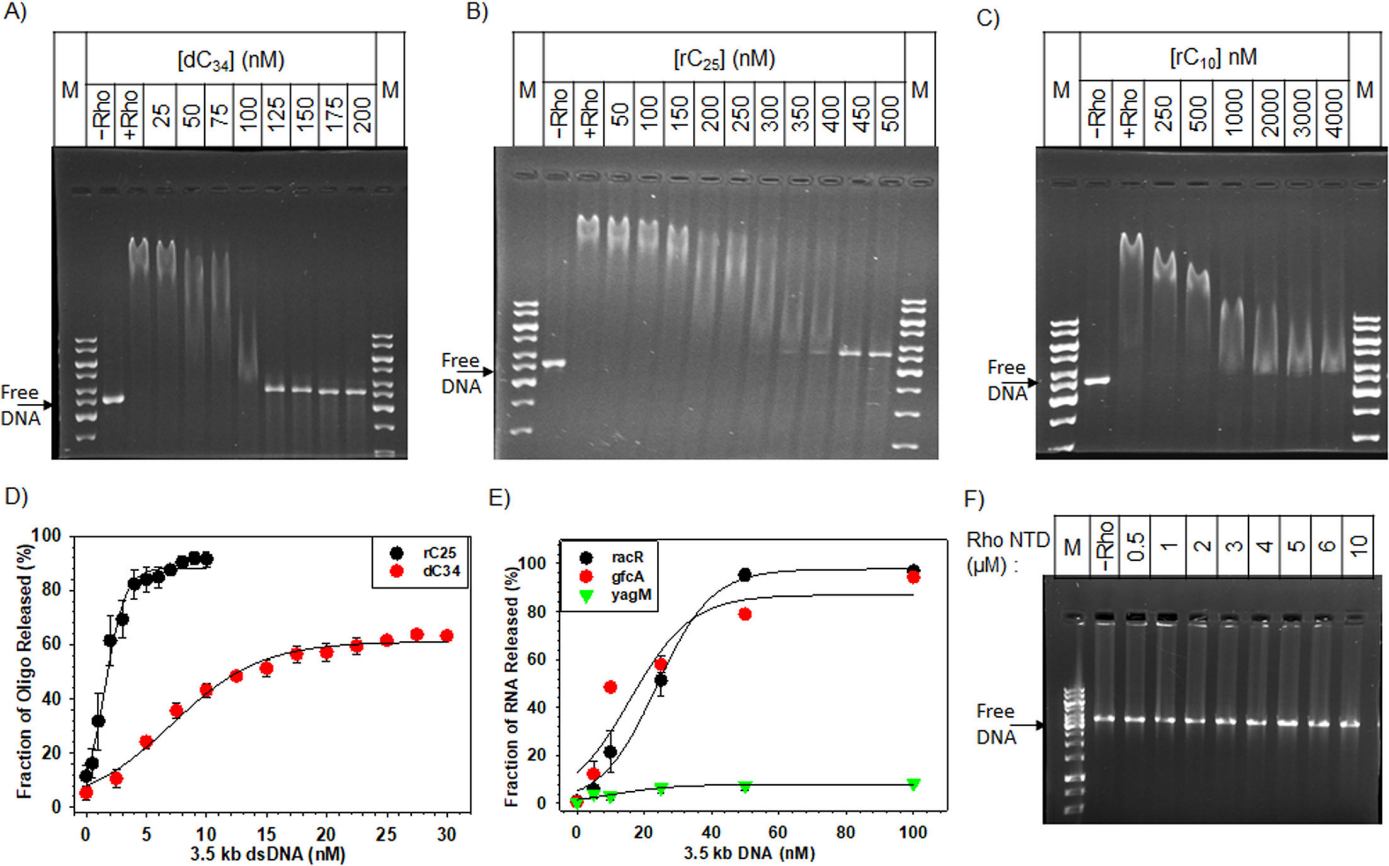
Competition between 3.5 kb dsDNA and ssDNA/RNAs for binding with Rho. A preformed Rho – dsDNA complex was challenged with the increasing concentrations of (**A**) dC_34_ (**B**) rC_25_ and (**C**) rC_10_. Concentrations of 3.5 kb DNA and Rho were 5 nM and 1 μM, respectively. In a reverse setup, complexes of Rho with ssDNA / RNAs are challenged with 3.5 kb dsDNA. Sigmoidal graphs showing the release of (**D**) small oligos (rC_25_ or dC_34_) or (**E**) long RNAs (racR, gfcA, yagM) from the complex with Rho in the presence of increasing concentrations of 3.5 kb dsDNA. The fractions release of ssDNA/RNA was plotted against the ratio of 3.5 kb dsDNA and the bound molecule. Error bars represent the SEM of three individual experiments. (**F**) EMSA with 3.5 kb DNA in the presence of increasing concentrations of monomeric Rho-–NTD fragment showed no shift in DNA position indicating that Rho-–NTD does not interact with DNA.

To assess the stability and specificity of the Rho–3.5 kb DNA complex, we challenged it with increasing concentrations of the RNAs or ssDNA known to bind with Rho (Figure 2 and Supplementary Figure S2; [20]. ssDNA, dC_34_ (34-mer poly dC), specifically binds to the Rho–NTD containing the primary nucleic acid binding site (PBS) [21]. ssDNA, dC_34_, competed out the 3.5 kb DNA from the Rho efficiently in the presence of 25-fold higher concentrations (125 nM; Figure 2A). On the other hand, the single-stranded small RNA competitors, rC_25_ (25-mer poly rC) and rC_10_ (10-mer rC oligo) that can interact with both the PBS and SBS of the Rho, exerted the same competition less efficiently at 90-fold (450 nM) and 400-fold (2000 nM) higher concentrations, respectively (Figure 2B, C). This suggests that the ssDNA oligo that binds specifically to the PBS can readily compete out the 3.5 kb dsDNA from the Rho. Consistent with the very weak binding of Rho to a 0.6 kb fragment (Figure 1A), this short dsDNA fragment also exerted very weak competition (Supplementary Figure S2A). These data also suggest that the Rho–PBS, a single-stranded nucleic acid-binding domain, might have the flexibility to accommodate a bulkier and rigid dsDNA. It should be noted that the dimension of the central channel of Rho (SBSs for the RNA) is such that it cannot accommodate a bulkier ds nucleic acid [PDBs: 3ICE]; hence, it is unlikely a long dsDNA would bind in this channel [22].

Next, we complexed Rho with different RNA and ssDNA molecules and challenged the complex with increasing concentrations of 3.5 kb dsDNA. We observed that the Rho–rC_25_ complex was competed out by an equimolar amount (5 nM) of the 3.5 kb dsDNA (Figure 2D and Supplementary Figure S2C. In contrast, a 6-fold (30 nM) excess of 3.5 kb dsDNA was able to compete up to a maximum of 60% dC_34_ suggesting equilibration between ssDNA and dsDNA for binding with Rho for the same PBS (Figure 2D and Supplementary Figure S2B). Challenging the Rho-longer RNA (RNA corresponding to the *racR* and *gfcR*) complexes required a 10-fold excess (50 nM) of the 3.5 kb dsDNA. The Rho–yagM RNA complex could not be competed out by the dsDNA (Figure 2E and Supplementary Figure S2D). This suggests that the dsDNA cannot compete with the stable interactions of longer RNAs that bind to both the six PBSs and an SBS of Rho. It should be noted that yagM is one of the strongest terminators of Rho-dependent termination [24].

Next, we checked whether dsDNA binds to the 130 amino acids monomeric Rho–NTD fragment that contains a single Rho–PBS and is capable of binding to ssRNA [3,4,23]. We analyzed the binding of 3.5 kb DNA with increasing concentrations of the monomeric NTD fragment of Rho (Rho–NTD; Figure 2F). Interestingly, even at a high concentration (10 μM; 10 times more than what was used in Figure 1 for the full-length Rho binding), the Rho–NTD with a single PBS was unable to bind with the dsDNA (Figure 2F) suggesting that the stability of the Rho–DNA interactions was due to the occupancy of the other PBSs of a hexameric Rho, which was absent in the monomeric Rho–NTD fragment and also likely due to the aggregated conformation of the hexameric Rho on the surface of the DNA. However, our data do not unequivocally indicate that all six PBSs of a Rho hexamer are simultaneously occupied in a Rho–dsDNA complex.

### Effect of dsDNA bound to Rho–PBS on its ATPase activity

**Figure 3:**
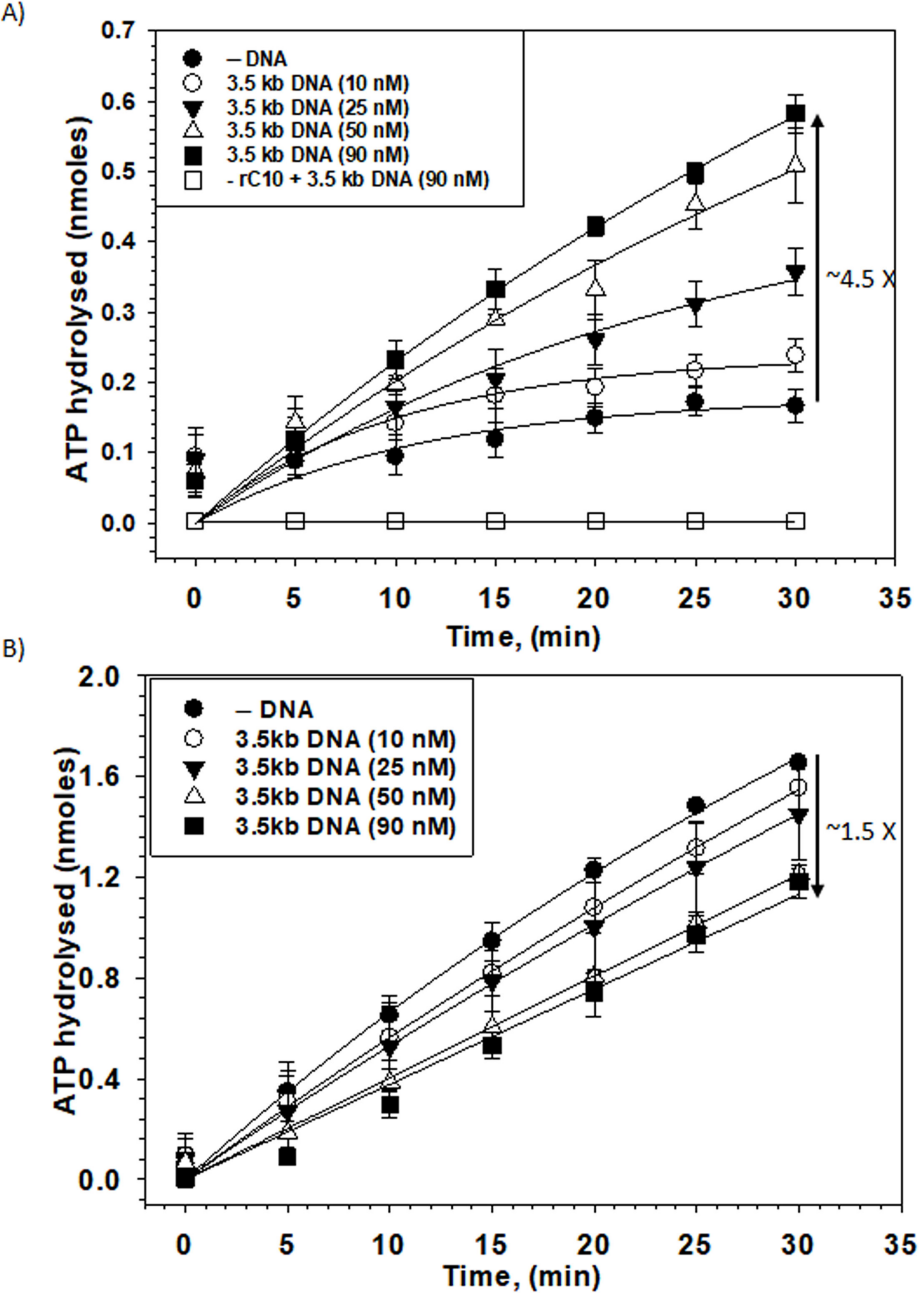
Effect of 3.5 kb dsDNA on the ATPase activity of Rho*.* (**A**) Rho ATPase activity in the presence of rC_10_ as a cofactor increases in the presence of increasing concentrations of dsDNA. The dsDNA does not induce ATPase activity in the absence of rC_10_. (**B**) ATPase activity of Rho as induced by *λtr1* RNA decreases in the presence of increasing concentrations of 3.5 kb dsDNA. Each point represents the average of three individual experiments with SEM as the error bar. Plots were fitted to the exponential rise equation using SigmaPlot 15.

As Rho possesses RNA-dependent ATPase activity, we examined the effect of dsDNA bound to its PBSs on the ATPase activity in the presence of different RNAs as co-factors. We used a small RNA oligo rC_10_ to check the ATPase activity of Rho. This oligo binds to the SBS of Rho but cannot elicit Rho’s ATPase activity by itself. However, this activity can be stimulated in the presence of an ssDNA oligo at Rho’s primary binding site (PBS) [21]. We hypothesized that if the 3.5 kB dsDNA binds to the Rho–PBS, it would stimulate rC_10_-induced ATPase activities like ssDNA oligos. rC_10_ oligo alone could elicit hydrolysis of only ~5% of the ATP molecules with a slow rate of hydrolysis (~4 pmoles/min; Supplementary Figure S3A). With the increasing concentrations of the 3.5 kB DNA, rC_10_ oligo could induce hydrolysis of ~23% of the ATP molecules (4.5-fold higher with 90 nM of dsDNA) after 30 minutes with a significantly higher hydrolysis rate (18 pmoles/min; Figure 3A and Supplementary Figure S3A). This suggests that similar to ssDNA [17], dsDNA could also stimulate the rC_10_-induced ATP hydrolysis process, and most likely it exerts this being bound to the PBS of Rho imparting similar allosteric conformational changes at the Rho–SBS-like ssDNA (Figure 3A).

Unlike a small RNA oligo, long RNAs can interact with both the PBS and SBS of Rho simultaneously and can induce ATPase activity on their own. We measured the *λt_R1_* RNA-induced Rho ATPase activity in the absence and presence of increasing concentrations of the dsDNA. We observed a dsDNA concentration-dependent reduction (~1.5 fold) in the ATPase activity of Rho as well as a reduction in the rate of ATP hydrolysis (from ~54 pmoles/min to ~41 pmoles/min; Supplementary Figure S3A), which could be due to the direct competition between the dsDNA and the RNA for the same Rho–PBS (Figure 3B). The Rho–dsDNA (PBS)–RNA ternary complex having dsDNA at the PBS and the *λt_R1_* RNA at the SBS is less efficient in ATP hydrolysis than the Rho*–λt_R1_* RNA binary complex. These results suggested that a 3.5 kb dsDNA at the PBS could induce the ATPase as well as a translocase–competent complex of Rho [25] allosterically albeit with a lesser efficiency.

### Effect of dsDNA on transcription termination activity of Rho

**Figure 4:**
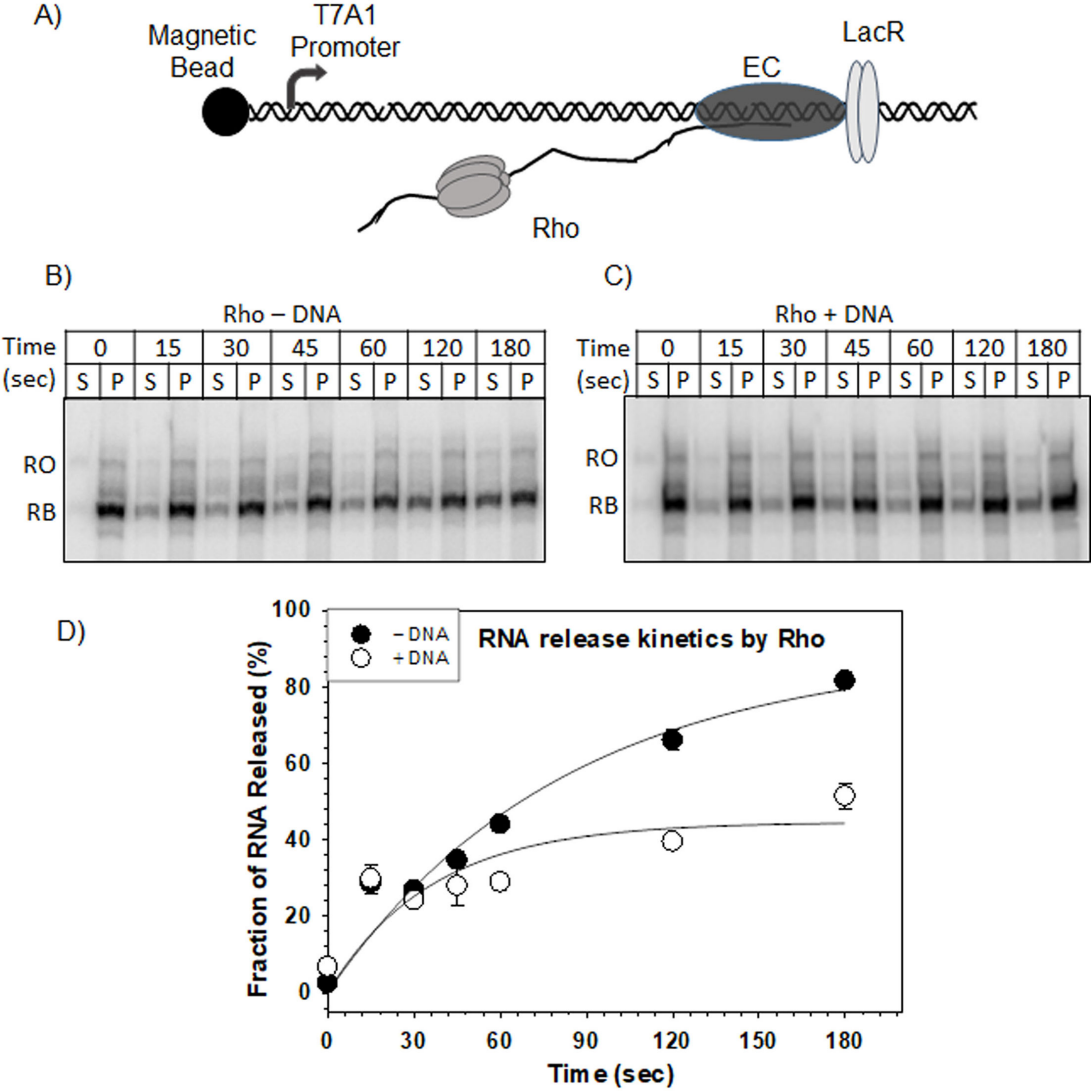
Effect of 3.5 kb dsDNA on the transcription termination activity of Rho*.* (**A**) Schematic representation of the EC stalled by the lac repressor (LacR) bound on a transcription template immobilized on a magnetic bead. Rho loading onto the nascent RNA to terminate the transcription is also shown. Autoradiograms showing the kinetics of Rho-dependent transcription termination of ECs stalled at LacR in the absence (**B**) and in the presence (**C**) of dsDNA. Rho was pre-bound to the 3.5 kb dsDNA before adding to the reaction. The transcripts that reached the end of the template are shown as run-off (RO), while those blocked by LacR are suggested as RB (Road-blocked). The fraction of RNA released by Rho was calculated from the intensity of bands at a roadblock (RB) as 2 S/ [S + (S + P)] where S represents half of the supernatant and P represents the half fraction of the supernatant along with the pellet fraction. (**D**) The graph shows fractions of RNA released by Rho in the presence or absence of dsDNA plotted against time. Curves were fitted to the exponential rise equation using SigmaPlot. Data represents an average of three replicates with error bars representing SEM.

Next, we examined the effect of Rho–dsDNA complex formation on the transcription termination by Rho. To monitor the effect of pre-bound Rho to 3.5 kb dsDNA, we first formed a stalled transcription EC at a lac repressor (LacR)–road block (RB) site downstream of a Rho terminator, *trpt’*. This EC was initiated from a T7A1 promoter, and the DNA template was immobilized on a magnetic bead (Figure 4A). We added free Rho and Rho complexed (prebound) with 3.5 kb dsDNA to the stalled EC in the presence of ATP and measured the efficiency and time course of Rho-dependent RNA release from this stalled EC (Figure 4B and C). We observed that the Rho prebound to the dsDNA template added *in trans* released RNA less efficiently and with a slower rate compared to that was observed with a free Rho (Figure 4D). In these experiments, concentrations of Rho and the 3.5 kb DNA were kept such that concentrations of free Rho were negligible. However, even if there are free DNA molecules present, to date, no mechanism is known by which a free linear dsDNA can affect the RNA release process from a stable RB EC. Therefore, we concluded that in this experimental setup, Rho might be scavenged out by a long stretch of free dsDNA from the process of transcription termination subjecting it to function with lesser efficiency.

### Rho overexpression and its impact on *in vivo* transcription

**Figure 5:**
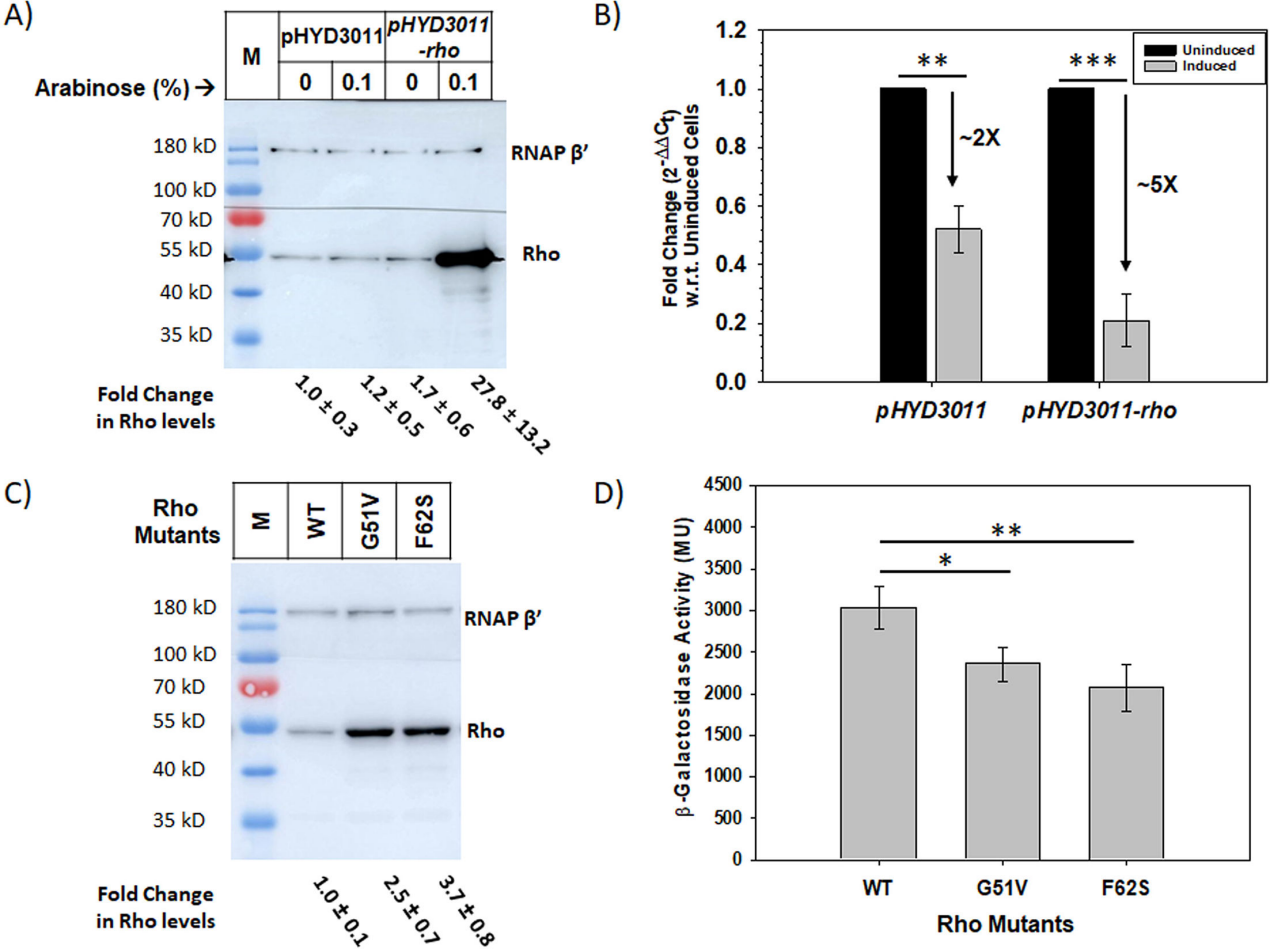
Effect of Rho overexpression on *in vivo* transcription. (**A**) Western blot showing the levels of Rho protein in *E. coli* strain RS1993 carrying the plasmids pHYD3011 or pHYD3011-*rho* in the presence or absence of 0.1% arabinose. The Rho bands in the empty vector or uninduced cell lanes represent Rho expressed from the chromosomal *rho*. Expression of Rho was normalized with the levels of RNAP β’ as a loading control. The fold changes in Rho levels are indicated below each lane. (**B**) RT-qPCR of *lacZ* genes under indicated conditions. The reduction of the *lacZ* RNA levels is indicated by downward arrows. The cells carrying empty plasmid showed a 2-fold reduction in *lacZ* expression which further reduced to 5-fold in the cells overexpressing Rho. The changes in *lacZ* expression were found significant by the Student’s *t*-test as indicated by *P < 0.05, **P < 0.01, ***P < 0.001, ****P < 0.0001. (**C**) Western blot showing the levels of Rho in different mutants. The level of RNA polymerase subunit in each of the strains was shown as loading controls in both the blots. The values below the blot represent the Rho level in cells carrying the Rho mutants compared to those carrying the WT Rho. (**D**) Bar diagram showing the β-galactosidase activity as the indicator of the *lacZ* expression from *P_lac_-lacZ* cassette in RS1993*Δrho* carrying the Rho WT or mutants. All values represent average data obtained from three individual experiments with error bars representing standard deviation. The differences in the β-galactosidase activities were found to be significant by Student’s *t*-test., as indicated by *P < 0.05, **P < 0.01, ***P < 0.001, ****P < 0.0001. The Standardstandard deviations indicated in (A) and (C) were obtained from three independent replicates. All the raw data used in these figures are shown in Supplementary Tables S3 and S4 Tables S3 and 4.

High-density nonspecific binding of Rho molecules to the chromosomes might affect the DNA-dependent process like transcription. Therefore, we next analyzed the *in vivo* expression level of *lacZ* in the presence of higher concentrations of Rho in an *E. coli* MG1655 strain. To elevate the concentration of Rho in *E. coli* RS1993 (*E. coli* MG1655 *Δlac Δrac P_Lac_-lacZ*), we over-expressed Rho from an arabinose-inducible vector pHYD3011 (a modified pBAD18 vector carrying *MCS* and *RBS* of the pET vector; [26]). Western blots of this strain in the presence of 0.1% arabinose revealed that *rho* cloned in pHYD3011 vector (pHYD3011-*rho*) expressed at a 27-fold higher level (Figure 5A). We checked the expression of *lacZ* mRNA in this strain in both the presence of physiological levels (in the absence of arabinose) and 27-fold higher levels of Rho (in the presence of 0.1% arabinose) by RT-qPCR assays. These assays revealed that the expression of *lacZ* mRNA was significantly reduced in the presence of high concentrations of Rho (Figure 5B). It should be noted that the presence of 0.1% arabinose itself reduces (2-fold) lacZ expression, and it is further reduced (5-fold) when a high level of Rho is present. Since the *P_lac_-lacZ* cassette inserted in the chromosome is devoid of the Rho-dependent terminator, we concluded that the enhanced reduction in *lacZ* transcript levels in these cells might be due to the inhibition of transcription resulting from the binding of Rho to this cassette.

In the above study, we increased the level of Rho unnaturally using an inducer, which could create artifacts. So, we next used two Rho mutants, G51V and F62S, with naturally higher expression levels compared to the WT Rho (Figure 5C) and checked the *lacZ* expression by measuring the β-galactosidase activities. Their higher expression levels occur because of failure in the Rho autoregulation control mechanism due to the presence of *rut* sites preceding the transcribing region of the Rho gene [27]. These two Rho mutants were capable of binding to the linear as well as plasmid DNA like the WT. However, the F62S Rho has moderately weaker affinity compared to the G51V Rho (Supplementary Figure S1B–D and Table S1B). Rho F62S was earlier shown to be defective in ssRNA binding [27]. They are also defective in transcription termination *in vivo* [27]. *E. coli* strain RS1993 (*E. coli* MG1655 *Δlac Δrac pLac-lacZ*) transformed with pCL1920 plasmid expressing either WT or Rho mutants (G51V and F62S) was used for the measurement of β-galactosidase activities. These mutants showed a significantly lower value of the β-galactosidase activity from the *P_lac_-lacZ* cassette inserted in the chromosome suggesting a reduced level of *lacZ* transcription from the lac promotor (Figure 5D). This reporter cassette in RS1993 is devoid of the rho-dependent terminator; therefore, we concluded that the reduction in β-galactosidase levels is due to reduced transcription resulting from the Rho binding to the DNA.

### Demonstration of *in vivo* interaction of Rho and DNA

Our results so far suggested that Rho can bind to dsDNA *in vitro* and this binding can affect its ATPase as well as termination functions. *In vivo*, bacterial chromosome might function as a dsDNA substrate for Rho, binding to which could affect the physiology of Rho. Hence, we probed the *in vivo* localization of Rho and its presumed interaction with the bacterial chromosome. We used the *E. coli* MG1655*Δrho* strain transformed with a plasmid expressing Rho fused with mCherry at its N-terminal via a linker (Supplementary Figure S4A, which produces red fluorescence on excitation at 561 nm. This construct was tested *in vivo* for its expression of the fusion protein by Western blot (Supplementary Figure S4B). The functional activity of the Rho–mCherry fusion was checked by its ability to support the growth of the bacteria (Supplementary Figure S4C). The strains expressing Rho–mCherry fusion protein showed a prominent band at ~74 kDa corresponding to Rho–mCherry fusion protein (Supplementary Figure S4B) and exhibited normal growth activity of the bacteria (Supplementary Figure S4C).

**Figure 6:**
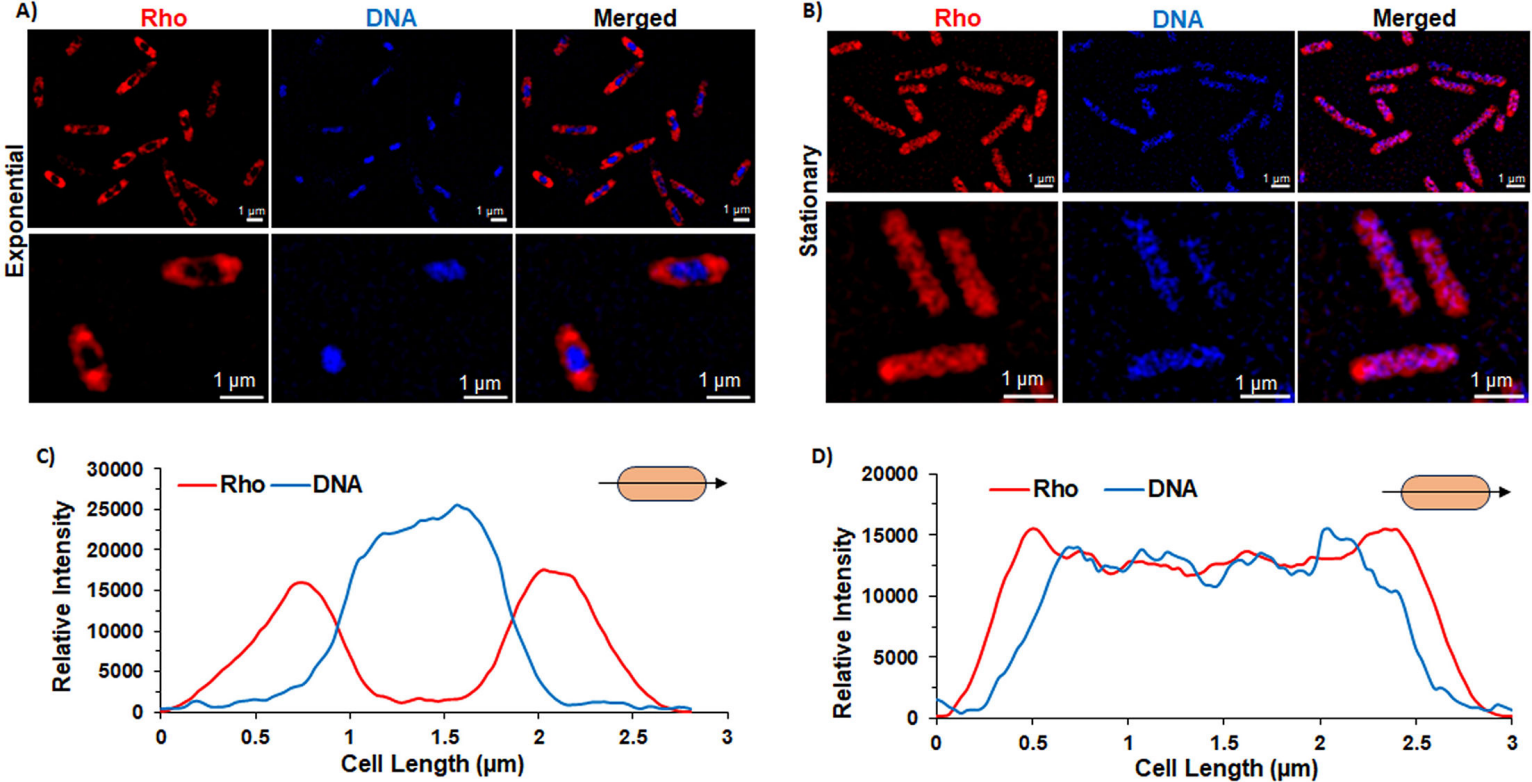
Cellular localization of Rho at different growth stages. Super-resolution microscope images of *E*. *coli* MG1655 *δrho* strain carrying plasmid pRS2264 expressing mCherry-–Rho at exponential growth phase (**A**) showing red signals of mCherry-–Rho at the poles and along the membrane. The blue signal is the chromosomal DNA stained with DAPI. (**B**) Super-resolution microscope images of mCherry-–Rho and the DAPI-stained chromosomal DNA at the stationary phase. Purple spots/patches in the merged panel are indicative of DNA-bound Rho (**C**) and (**D**) Aaverage intensity profile plots (obtained from 30 cells) along the central longitudinal axis from the indicated growth stages. The X-axis measures the length of the cell along the longitudinal axis as shown in the cartoons. The white line scale bar indicates 1 µm for all panels.

The MG1655 *Δrho* strain expressing Rho–mCherry fusion protein from pCL1920 plasmid was grown until OD_600nm_ = 0.4 (log phase) or 1.0 (stationary phase) in a minimal medium. We viewed Rho–mCherry and DNA (chromosome) inside the cells simultaneously by SIM super-resolution microscopy using 405 nm (for DAPI-stained chromosome) and 561 nm (for mCherry) laser excitations, respectively (Figure 6). In general, Rho–mCherry was seen to be distributed primarily at the poles and along the membranes and was excluded from the central part of the cells that was occupied by the DAPI-stained bacterial chromosome in the log phase cultures (Figure 6A). The intensity profile of the two colors along the longitudinal axis of a cell clearly distinguishes the residing zones of the Rho (peaks at the poles) and the chromosome (central part of the cell; Figure 6C). This suggests that during the log phase, Rho spends much less time near or bound to the chromosome. However, at the stationary phase (Figure 6B), the mCherry–Rho was observed to be more distributed randomly in the cytoplasm of the cell, a much smaller number of molecules were located at the poles, and, likely, a significant fraction of these randomly distributed Rho molecules was spread over the bacterial chromosome (see the purple patches/spots in the merged panel of Figure 6B). In this stationary phase, the peak intensity profiles of the red (mCherry) and the blue colors (DAPI) considerably overlapped along the longitudinal axis of the cell (Figure 6D). These data suggested that in the presence of very low-level transcription activities during the stationary phase, Rho molecules lose specific interactions at the poles and move randomly in the cytoplasm, of which a significant fraction could “rest” on the surface of the bacterial chromosome using its DNA-binding ability.

## Discussion

The bacterial transcription termination factor Rho is an RNA helicase that is capable of translocating along the RNA using the energy from ATP hydrolysis. Earlier *in vitro* studies showed that small oligonucleotides bind to the N-terminal PBS of Rho [20,21]. So far, no detailed analysis of the DNA-binding properties of Rho as well as the functional consequence of such binding has been undertaken. Here, we showed that Rho binds to linear long dsDNAs and also to the circular plasmids nonspecifically via its PBS (Figure 1). Both the NHB and OB-fold components of PBS might be involved in this binding. This Rho–PBS dsDNA interaction could be competed efficiently by ssDNA, dC_34_, and also by single-stranded RNAs at much higher concentrations (Figure 2). The presence of long dsDNA (3.5 kb) at the PBS isomerizes Rho into a closed form competent for ATPase activities at the central channel (Figure 3). The Rho prebound to a long DNA affects its transcription termination efficiency *in vitro* (Figure 4). Such DNA-bound Rho molecules also exert negative effects on *in vivo* gene expression (Figure 5). *In vivo,* a significant fraction of Rho molecules was observed to be spread over the bacterial chromosome in the stationary phase when bacterial transcription is usually very low. And, in the mid-log phase, Rho molecules were seen near the poles and along the membrane, which suggests that Rho spends much less time near the bacterial chromosome in this phase (Figure 6). We propose that Rho molecules not engaged in the transcription termination process during the stationary phase could use the bacterial chromosome as a “resting surface” before getting transferred directly to the *rut* sites on the nascent RNA as and when the necessity arises [28,29].

In two recent structural studies, hyper-oligomerization of Rho was observed under *in vitro* conditions when it is bound to a bacteriophage protein Psu [30] or when it is bound to stress-inducing molecules like ppGpp or ADP [31]. We envisage that *in vivo* the hyper-oligomerization states of Rho could be formed aided by the surface provided by the bacterial chromosome in the stationary phase or under stress.

During the rapidly growing stage (mid-log phase), most of the transcripts are coated with ribosomes to form a structure called polysome that is engaged in translation, and Rho molecules are generally excluded from the RNA. The expression of Rho is constitutive throughout all the growth phases [32]. Hence, in the mid-log phase, most of the Rho molecules are likely to stay “idle” without engaging in the transcription termination processes. Our evidence of the Rho localized to the poles of the cell instead of DNA-bound transcription ECs is the “idle” Rho molecules. We also speculate that the specific interactions of Rho at the poles keep it away from accidentally loading onto the nascent RNA and initiating unwanted transcription termination when the demand for protein synthesis is very high at the log phase. It should be noted that our microscopy images only gave a snapshot view of the localization Rho where it spends the majority of the time. These images did not reveal the dynamic behavior of Rho recruitment to the transcription complexes during the log-phase growth when it is only required to suppress the unwanted pervasive or anti-sense transcriptions or when there is an occasional failure of ribosome loading onto the mRNA.

However, a high density of Rho molecules on bacterial DNA is likely to affect the binding and movement of stationary-phase-specific DNA-binding proteins and the processes associated with them, which could affect cellular physiology. Due to the presence of a feedback control mechanism [33,34], chromosomal Rho level never become very high under normal growth conditions. In an earlier study [27], it was revealed that this feedback control fails to operate in the case of a few Rho mutants that have weak RNA-binding properties. It is expected that in those cases high-density Rho patches could be present on the chromosome. It should be noted that in *M. tuberculosis*, overexpression of Rho is lethal to the cells [35].

Rho is engaged in many physiological processes [1]. Its pleiotropic effect mainly arises from its nonspecific RNA-binding and helicase properties. From the microarray as well as transcriptome analyses [6,9,36], it was evident a significant number of genes were downregulated when the transcription termination activities of Rho were impaired. We surmise that a major portion of this downregulation could have arisen from the nonspecific DNA binding of Rho under the conditions when Rho is not engaged in the transcription termination process. Hence, Rho’s DNA-binding property might also indirectly contribute to its pleiotropic effect on the physiology of the cells.

## Materials and methods

### Materials

NTPs were purchased from GE Healthcare. We obtained [γ-^32^P]ATP, [α-^32^P]UTP, and [α-^32^P]CTP at 3000 Ci/mmol from Jonaki, BRIT, Hyderabad. *E. coli* RNA polymerase holoenzyme, T4 PNK, and T4 DNA ligase were from New England Biolabs (NEB). Antibiotics, IPTG, lysozyme, DTT, and bovine serum albumin (BSA) were obtained from the United States Biochemical Corporation (USB). DNA oligos and small RNA oligos were synthesized by Eurofins Genomics India Pvt. Ltd. Long RNAs (racR, gfcA, yagM, and λtR1) were synthesized using AmpliScribe™ T7-Flash™ Transcription Kit from Lucigen as described in [18]. Taq DNA polymerase was obtained from Roche Applied Science. Ni-NTA agarose beads for protein purification were from Qiagen. All other chemicals were procured from Sigma or Thermo Fisher Scientific Limited. Plasmids and DNA oligos used in this study are listed in Supplementary Table S2.

### DNA preparation for Rho interaction

DNAs for *in vitro* Rho interaction were prepared by PCR amplification. Smaller DNAs (0.6 and 1.3 kb) were PCR amplified with primer pair RS58/RS-RK23B and RS58/RSRK1, respectively, using plasmid pRS22 as a template. Another 0.6 kb DNA was amplified from *E. coli* MG1655 genomic DNA (gDNA) using primer pair RS300/RS1744. DNAs of length 1.5 kb, 2.0 kb, and 3.5 kbp were amplified from the *E. coli* MG1655 gDNA using primer sets RS1744/RS84, RS1743/RS555, and RS1743/RS1744, respectively.

### Gel shift assays

The interaction between Rho and DNAs of different lengths was examined by gel shift assay. Here, 5 nM DNA was mixed with different concentrations of Rho in T buffer (25 mM Tris-Cl pH 8.0, 50 mM KCl, 5 mM MgCl_2_, 1 mM DTT, and 1 mM ATP) and incubated at 37°C (or at 4°C when indicated) for 15 minutes. Samples were electrophoresed on 0.8% agarose gel in 0.5× TBE and visualized by ethidium bromide staining. The fractions of bound DNA were measured using ImageQuant software and were plotted against the concentration of Rho. The plots were fitted to a sigmoidal-binding model using SigmaPlot 15 to determine the *K*_d_ values. Rho binding to plasmids of different sizes was also examined similarly.

### Competition assay

To assess the competitive binding between dsDNA and ssDNA or RNA with Rho protein, first Rho–dsDNA complex was formed by incubating 5 nM dsDNA and 1 μM Rho in T buffer as above. The competitor molecules (0.6 kB dsDNA, ssDNA (dC_34_) or small RNAs (rC_10_ and rC_25_) were added in increasing concentrations and incubated further for 15 minutes. Reaction mixtures were electrophoresed on 0.8% agarose gel to check for release of 3.5 kb DNA.

In reciprocal experiments, 100 nM Rho was first bound to 5 nM of ^32^P-radiolabeled dC_34_ or RNAs (rC_25_, racR, gfcA, and yagM) which is then competed with increasing concentrations of 3.5 kb DNA. The release of RNAs or ssDNA was analyzed by electrophoresing on 4–8% gradient native PAGE in 0.5× TBE. The fractions of bound and released molecules were visualized by scanning using a Typhoon 9200 phosphor imager. The fraction of released RNAs or dC_34_ DNA was quantified by ImageQuant software and plotted against the concentration of dsDNA competitor added, and the binding isotherms were fitted to a sigmoidal model using SigmaPlot 15.

### ATPase assay

RNA-dependent Rho ATPase activity was measured by mixing 50 nM Rho in buffer containing 25 mM Tris pH 8.0, 50 mM KCl, 5 mM MgCl_2_, 1 mM DTT, 0.1 mg/ml BSA, 1 mM ATP, and a trace amount of γ-^32^P ATP. ATPase activity was induced by the addition of 1 μM rC_10_ or 200 nM *λtR_1_* RNA. Aliquots at different time points were collected, and the reaction was stopped with 1.5 M formic acid. Inorganic phosphates (Pi) released from the hydrolysis of γ-^32^P ATP were observed by separating them on polyethyleneimine TLC plates with 0.75 M KH_2_PO_4_ as mobile phase. The TLC plates were then exposed to phosphor imager plates and scanned with typhoon phosphor imager. The effect of dsDNA on ATPase activity was monitored by incubating Rho with different concentrations of dsDNA before activation of ATPase activity by RNA. The rate of ATP hydrolysis was calculated by plotting the concentrations of ATP hydrolyzed at specific time points against the time.

### *In vitro* transcription assay

To assess the effect of the addition of DNA on transcription termination by Rho, we performed *in vitro* transcription in the presence and absence of the dsDNA. The DNA template for *in vitro* transcription was generated by PCR amplification with primers RS83 and RS177 from the plasmid pRS106 resulting in a template with *trpt’* terminator fused with LacO sequences. The transcription initiation complex was formed by mixing 5 nM DNA template, 25 nM RNA polymerase in the transcription buffer (25 mM Tris HCl pH 8.0, 5 mM MgCl_2_, 50 mM KCl, 1 mM DTT, and 0.1 mg/ml BSA). To halt the RNAP near the end of the sequence, 300 nM LacR was added to the reaction and further incubated for 10 min. The transcription reaction was completed with the addition of 250 μM each of ATP, GTP, CTP, and UTP for 2 minutes. After washing the transcription assembly with transcription buffer, the transcription reaction was terminated by the addition of 50 nM Rho in the transcription buffer containing 1 mM ATP. To examine the effect of dsDNA on Rho activity, 50 nM Rho was pre-incubated with 50 nM of 3.5 kb DNA before adding it to the transcription mixture. At specific time points, half of the supernatant (fraction S) was collected to assess the released RNA from the template. The other half fraction of the supernatant along with the pellet fraction (fraction S + P) was subjected to phenol extraction to collect complete transcribed RNA. RNA samples were separated on 8% denaturing PAGE with 1× TBE at 1600 V for 2.5 hours. The gels were dried and exposed to phosphor imager films and scanned with Typhoon 9200 phosphor imager. Fractions of RNA release were calculated as 2 S/[S + (S + P)] and plotted against the time points.

### Construction and validation of Rho–mCherry fusion protein

Rho–mCherry fusion protein was expressed under the control of *rho* promoter from the plasmid pRS2264 generated by overlap extension PCR [37] where *mCherry* gene with a 45 bp linker was inserted upstream of *rho* gene in plasmid pCL1920. As a control to check for the free mCherry inside the cell, plasmid pRS2246 carrying *mCherry* gene expressed under the control of *rho* promoter in plasmid pCL1920 was constructed similarly.

To check the expression of the fusion protein, *E. coli* MG1655 was transformed with plasmid pRS2264, followed by deletion of chromosomal *rho* by P1 transduction. The deletion strain carrying pRS2264 was grown until OD_600 nm_ reached 0.4. The cells from the 200 μl culture were harvested and suspended in 50 µl of 1× Laemmli buffer. Proteins in the culture were analyzed by separating on a 10% SDS-PAGE followed by a Western blot. The blot was probed with an anti-mCherry antibody (AB167453, Abcam) as the primary antibody, followed by an anti-Rabbit IgG HRP-conjugated antibody (A0545, Sigma-Aldrich Chemicals Private Limited) as the secondary antibody. The proteins present in the lysates were detected with ECL prime Western blotting detection reagent (Amersham) using the ImageQuant™ Chemiluminescent Imaging System, LAS 500. *E. coli* MG1655 *Δrho* transformed with pRS317 expressing only Rho protein and *E. coli* MG1655 WT transformed with pRS2246 expressing only mCherry protein were used as controls.

To assess the effect of fusion protein on the growth of *E. coli*, overnight cultures of *E. coli* MG1655 *Δrho* transformed with pRS2264 expressing Rho-mCherry fusion protein and pRS317 expressing only Rho protein were sub-cultured in a microtiter plate and growth profiles were obtained by incubation in a SpectraMax M5 microtiter plate reader (Molecular Devices, United States).

### Rho–DNA co-localization analysis with fluorescence microscopy

For these studies, *E. coli* MG1655 *Δrho* strains transformed with plasmid pRS2264 expressing Rho–mCherry were at first grown in LB medium with appropriate antibiotics for 16 hours at 37°C. Here, 1 ml of this primary culture was washed twice with minimal medium supplemented with 0.0001% vitamin B1, 1 mM MgSO_4_, and 0.2% glucose. In total, 1% of the primary culture was subsequently used for a secondary culture in the minimal medium till the growth reached the exponential phase (OD_600 nm_ ~ 0.4) or stationary phase (OD_600 nm_ ~ 1.0). In minimal media, the stationary phase reached ~0.9 OD_600_ (data not shown). An aliquot of 4 ml of exponential phase cells and 1 ml of stationary phase cells were collected and proceeded further for sample preparation. Cells were washed thrice with 1× PBS (pH 7.4) and fixed with 4% paraformaldehyde (in 1× PBS) for 30 minutes on ice. Following another three washes with 1× PBS to remove excess fixing agents, cell pellets were resuspended in 100 µl of DAPI solution (10 µg/ml) and incubated at 37°C for 30 minutes. Samples were washed twice with 1× PBS to remove excess DAPI solution and finally dissolved in 200 µl of 1× PBS containing 20% glycerol as mounting medium. A 5 µl sample was spotted on a clean glass slide and covered with a poly-l-lysine-coated coverslip to immobilize the cells. The slides were sealed with nail polish and proceeded for imaging.

The cells were observed under a super-resolution microscope ELYRA 7 (Zeiss LSM 980) with in-built lattice SIM techniques. The objective features a Plan-Apochromat 63×/1.4 Oil DIC M27 lens with a laser exciting at 561 nm for mCherry-tagged Rho fluorescence detection and 405 nm for DAPI-stained DNA fluorescence detection. The exposure time was 80 ms for 561 nm wavelength and 40 ms for 405 nm wavelength. The depth of the focus was 0.87 μm and 0.63 μm for mCherry and DAPI, respectively. All the captured images (16 bits) were further processed using Zen (version 3.10) software.

### Effect of Rho overexpression on *in vivo* transcription

We examined the effect of Rho overexpression on *in vivo* transcription in *E. coli* MG1655 *Δlac Δrac* carrying a *P_lac_-lacZ* cassette inserted in the chromosome by λRS45-mediated transduction (RS1993). We transformed this strain with plasmid pHYD3011 expressing *rho* under an arabinose-inducible *P_BAD_* promoter (pRS632). This vector pHYD3011 is a modified pBAD18 plasmid having *MCS* and *RBS* of pET vector for overexpression of target gene under the control of arabinose promotor [26]. The cells carrying empty vector pHYD3011 (pRS669) were used as control. The overnight cultures of RS1993 transformed with pHYD3011-*rho* (pRS632) or pHYD3011 empty plasmid (pRS669) grown in triplicates inoculated with three individual colonies were sub-cultured in LB broth in the absence or presence of 0.1% arabinose and were grown till mid-log phase (OD_600_ nm ~ 0.3–0.4). At this point, the cultures were divided into two fractions: one fraction was used to isolate RNA, and another was used to measure the Rho expression level by Western blot. The total RNA was isolated using RNeasy Plus Mini Kit (Qiagen) as per the manufacturer’s protocol. cDNA was generated using SuperScript™ III Reverse Transcriptase with random hexamers. The expression level of the *lacZ* gene was measured using BioRad CFX96 Real-Time PCR Detection System by amplifying the middle region of *lacZ* using the primer pair RS2461/RS2462 with the cDNA as a template. As an internal control, the expression level of RNAP β’ (*rpoC*) was also monitored by amplification of the *rpoC* region with primer pair RS2086/RS2087. The fold change (2^−ΔΔCt^) of *lacZ* expression in the induced cell was calculated from the values of threshold cycle (C_t_) using the following equations:


$$\Delta C_{t}=C_{t(target\;Gene)}-C_{t(reference\;gene)};\Delta \Delta C_{t}=\Delta C_{t(induced\;cells)}-\Delta C_{t(uninduced\;cells)}.$$

The significance of the results obtained was analyzed by Student’s *t*-test.

The levels of Rho overexpression in these samples were analyzed by Western blot using anti-Rho antibodies raised in rabbits as the primary antibody followed by anti-rabbit HRP-conjugated IgG as the secondary antibody. The protein levels were detected with ECL prime Western blotting detection reagent (Amersham) using the ImageQuant™ Chemiluminescent Imaging System, LAS 500. We also measured the levels of the RNAP β′ subunit in each sample as loading control by probing the same blot with antibodies against the RNAP β′ subunit.

### Effect of Rho mutants on bacterial physiology

We examined the effect of Rho mutants (G51V and F62S), which are defective in RNA binding at its PBS and hence are also defective in autoregulation of its expression resulting in 3- to 4-fold higher expression of Rho compared with the WT counterpart. We used *E. coli* strain RS1993 transformed with WT and mutant derivatives of Rho cloned in pCL1920. This vector expresses Rho from its promotor at a level comparable to the expression level of chromosomal *rho*. Following transformation, chromosomal *rho* was deleted by P1 transduction. As described above, the resultant strains were examined for the Rho expression levels by Western blot.

These strains were also analyzed for intracellular β-galactosidase activity as an indicator for lacZ expression by the Miller method [38,39]. Briefly, an overnight culture of these strains was subcultured in fresh LB broth and grown at 37°C till the mid-log phase (OD_600_ nm ~ 0.3–0.4). An aliquot of these cultures was mixed with Z-buffer (60 mM Na_2_HPO_4_, 40 mM NaH_2_PO_4_, 10 mM KCl, 1 mM MgSO_4_, and 50 mM β-mercaptoethanol) to make the final volume 1 ml. The cells were then lysed by the addition of 40 μl chloroform and 20 μl 0.1% SDS followed by incubating at 28°C for 5 minutes. The assay was initiated by the addition of 200 μl ONPG (4 mg/ml) and continued till the development of yellow color. The reaction was stopped by the addition of 500 μl 1 M Na_2_CO_3_, and the OD_420nm_ values were recorded after brief centrifugation. The β-galactosidase activity was calculated in Miller units (MUs) using the equation:


$$MU=1000\times OD_{420nm}/(T\times V\times OD_{600nm}),$$

where *T* is the reaction time (time between the addition of ONPG and Na_2_CO_3_) in minutes, *V* is the culture volume (in ml) taken for reaction, and OD_600nm_ is the absorbance of culture.

### Statistical analysis

For different assays in Figure 5A–D, average values were calculated from the results obtained from independent replicates (3–4), and error was calculated as standard deviation (SD) using the function STDEV.P in Microsoft Excel 2016. The unpaired two-tailed Student’s *t*-test using Microsoft Excel 2016 was performed to assess the statistical significance of the data obtained in the qPCR and β-galactosidase assays. The *P* values obtained were represented as *P < 0.05, **P < 0.01, ***P < 0.001, ****P < 0.0001. The raw data of these assays are given in Supplementary Tables S3 and S4.

## Supplementary material

online supplementary material 1.

## Data Availability

All the raw data used to prepare the final figures or tables are available upon request. This manuscript does not contain any dataset that must be deposited to any data bank.

## Open access

This article has been published open access under our Subscribe to Open programme, made possible through the support of our subscribing institutions, learn more: here https://portlandpress.com/pages/open_access_options_and_prices#conditional

## Abbreviations

EC: elongation complex
LacR: lac repressor
MU: Miller unit
NEB: New England Biolabs
NHB: N-terminal helix bundle
OB-fold: oligonucleotide binding fold
PBS: primary binding site
Pi: inorganic phosphates
RB: road block
RO: run-off
SBS: secondary binding site
SD: standard deviation
USB: United States Biochemical Corporation
ds: double-stranded
dsDNAs: double-stranded DNAs
gDNA: genomic DNA
