# Supplementary material for: DNA binding of an RNA helicase bacterial transcription terminator
Source: Biochem J. 2025 Jan 31;482(3):BCJ20240452. doi: 10.1042/BCJ20240452 (PMC12133298; doi:10.1042/BCJ20240452)
Supplement: online supplementary material 1. [file bcj-482-3-BCJ20240452-s001.pdf]

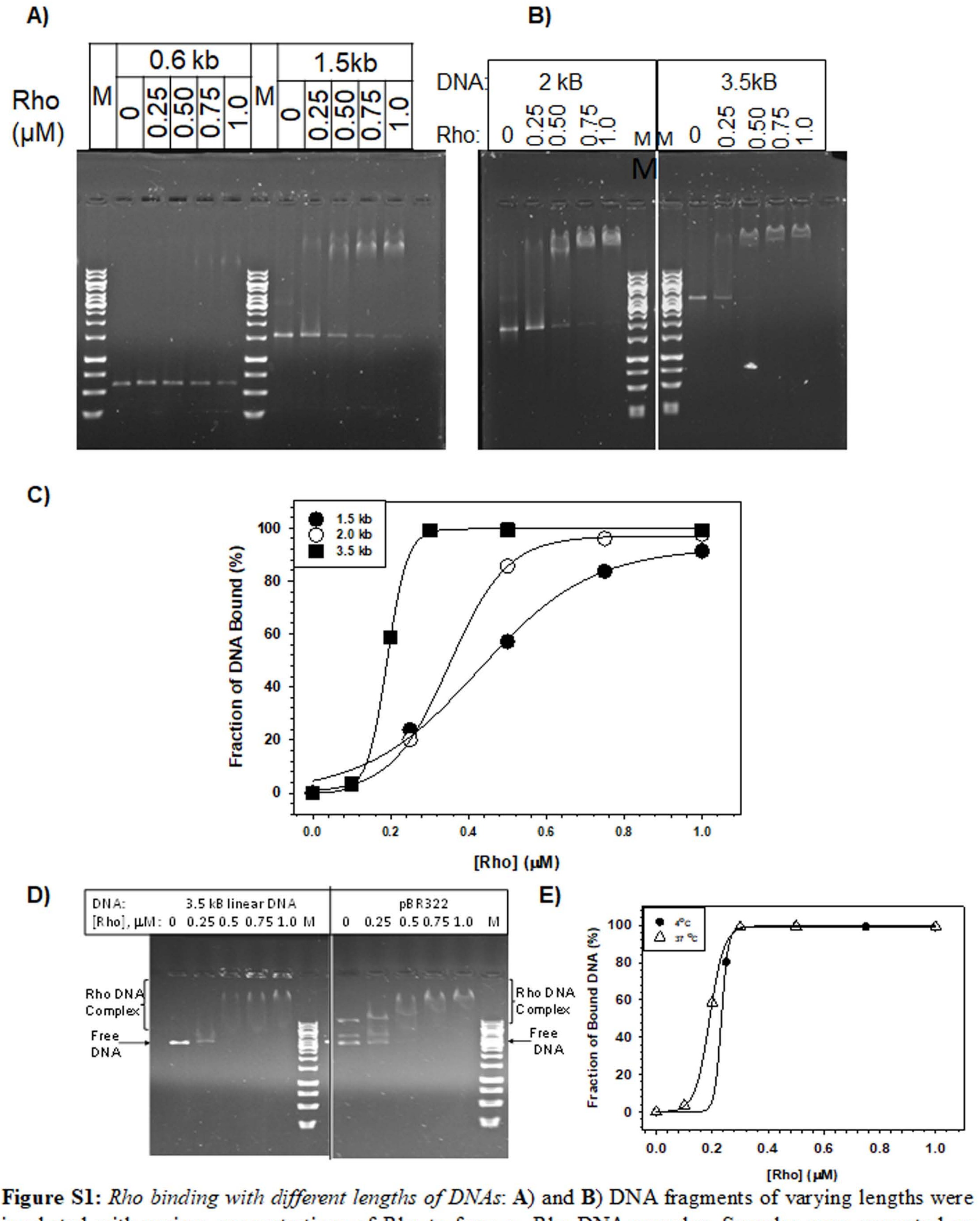

Figure S1: *Rho binding with different lengths of DNAs*: **A**) and **B**) DNA fragments of varying lengths were incubated with various concentrations of Rho to form a Rho-DNA complex. Samples were separated on agarose gel and visualized with ethidium bromide. In this figure, the 0.6 kB DNA was prepared from the gDNA of MG1655 by PCR using the primers RS300/RS1744, the sequence of which is different from the 0.6 kB fragment shown in Figure 1A. **C**) Fractions of bound DNA were estimated. Rho binds most efficiently with 3.5 kb DNA whereas 0.6 kb DNA had the least affinity. D) Gel-shift assays at 4°C with the 5 nM each of the indicated DNA. E) Fraction of linear 3.5 kb DNA bound with Rho at 4°C were calculated. Rho binding with DNA was more efficient at 4°C than that at 37°C.

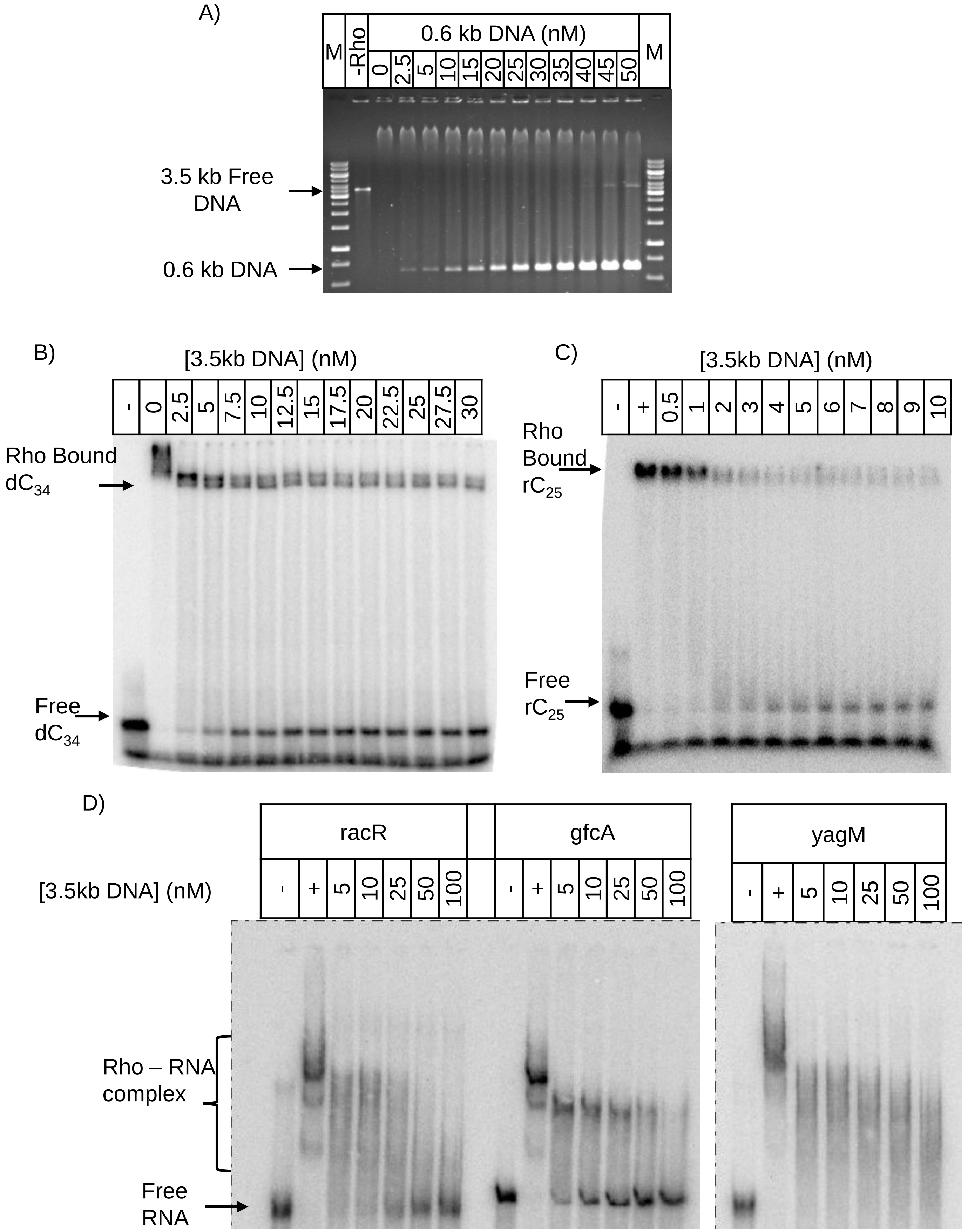

Figure S2: Dissociation of Rho complexed with indicated ds/ssDNA and RNA- A) Rho complexed with 3.5 kb DNA was challenged with increasing concentrations of 0.6 kb dsDNA amplified from plasmid pRS22 as indicated in the legend of Figure 1. Representative autoradiograms showing the extent of dissociation of the Rho-3.5 kB complexes with the B) dC_{34}, C) rC_{25}, and D) long RNAs (racR, gfcA, and yagM) bound to Rho upon competition with 3.5 kb linear DNA. Concentrations of DNA/RNA are measured in DNA or RNA ends.

A) RNA:

rC_{10}

[3.5 kB DNA]	Av. Rates of ATP Hydrolysis (pmol/min)
0	3.77
10 nM	4.99
25 nM	9.13
50 nM	14.73
90 nM	18.02

λt_{R1}

[3.5 kB DNA]	Av. Rate of ATP Hydrolysis (pmol/min)
0	54.5
10 nM	49.4
25 nM	46.6
50 nM	39.5
90 nM	41.1

B)

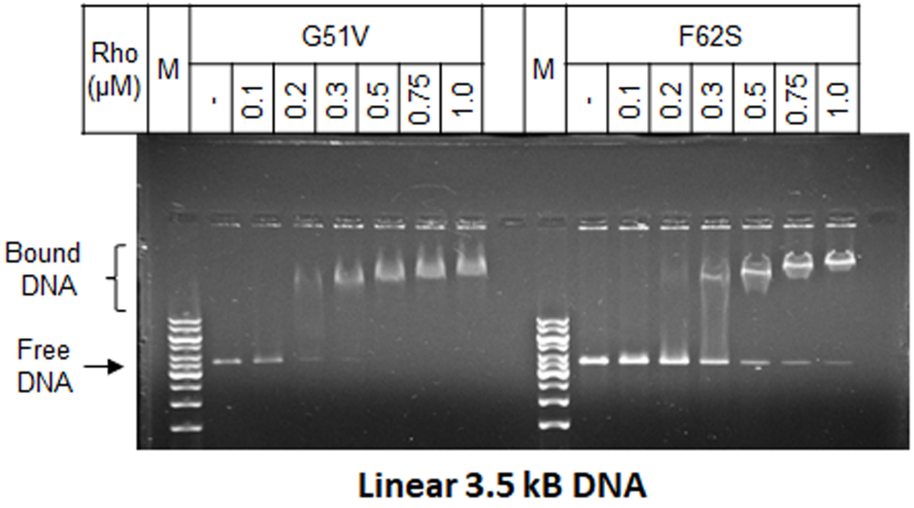

C)

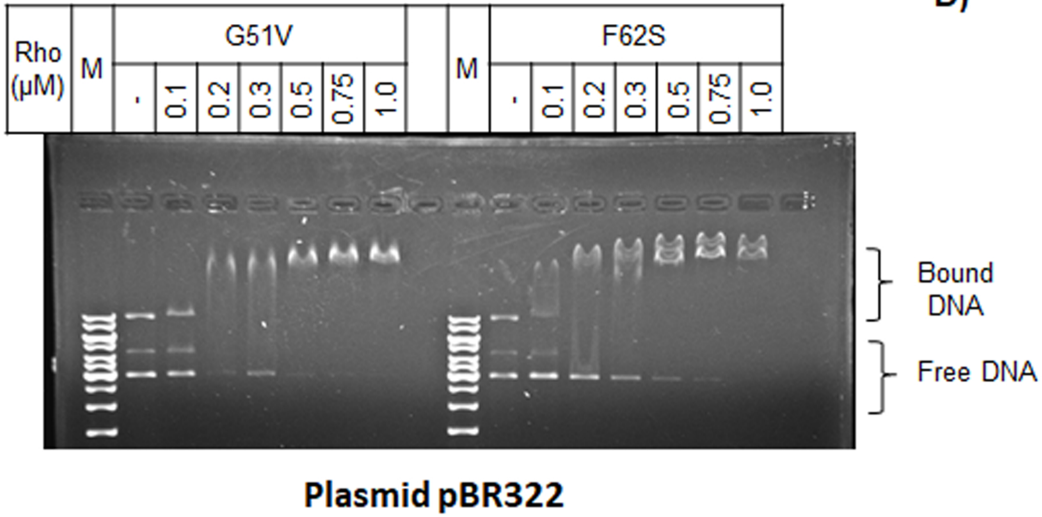

D)

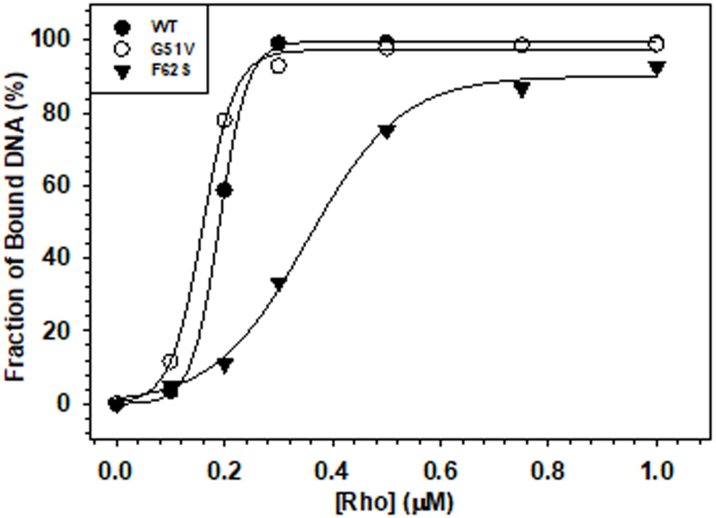

Figure S3: (A) Rates of ATP hydrolysis in the presence of indicated RNA co-factors obtained from the plots shown in Figure 3. EMSA of B) linear 3.5 kb DNA and (C) plasmid pBR322 with increasing concentrations of Rho G51V and F62S. (D) Fraction of linear DNA bound to Rho WT and mutants were plotted against the concentration of Rho to calculate the dissociation constants.

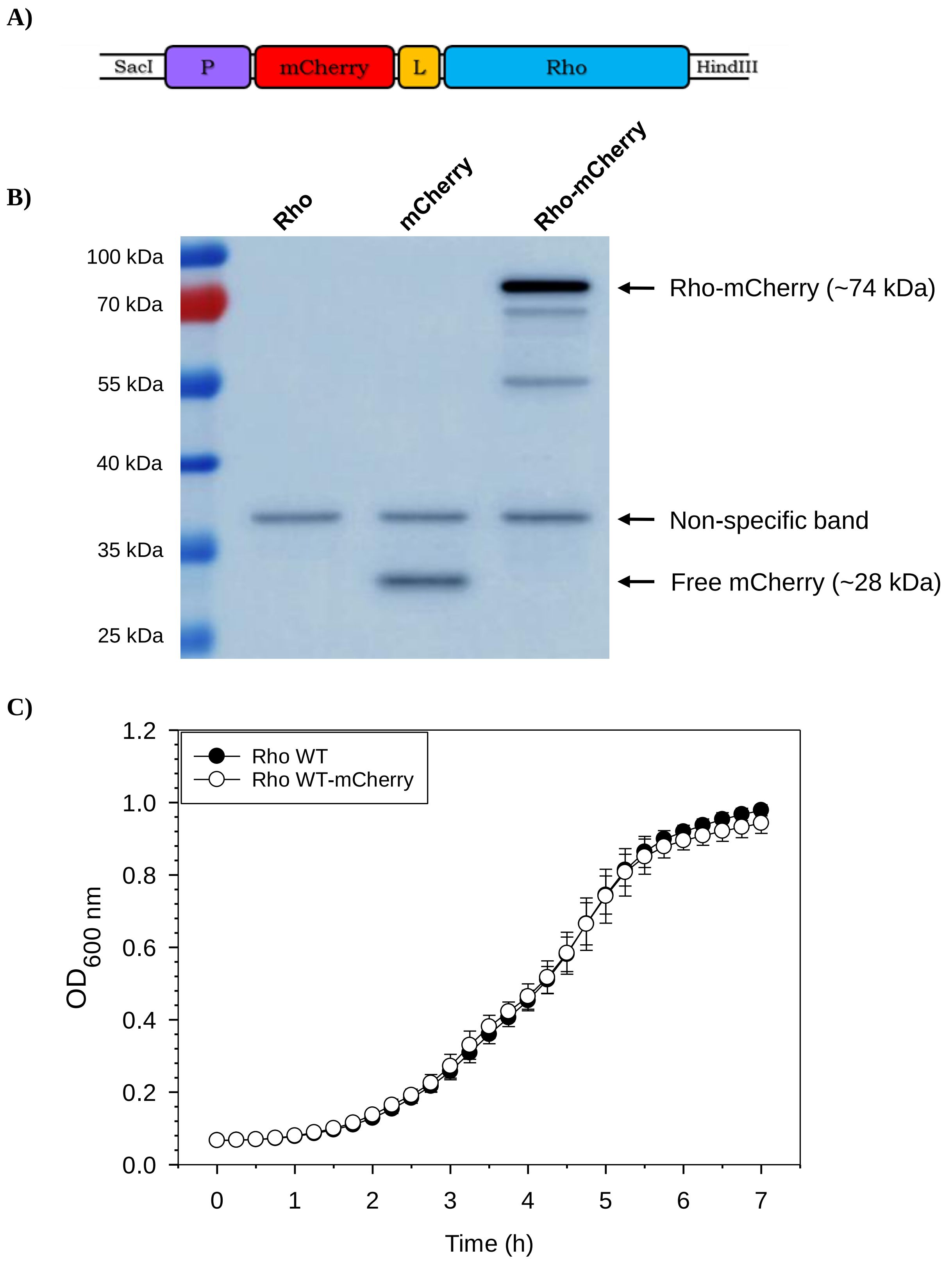

Figure S4: *Rho-mCherry fusion construct:* (A) mCherry tagged to the N-terminal of Rho under the control of Rho promoter (P) in a pCL1920 plasmid; A flexible linker (L) of 15 amino acids residue ([Gly-Gly-Gly-Gly-Ser]$_3$) is used in between mCherry and Rho. (B) Western blot of whole cell proteins from *E. coli* MG1655 strain expressing native Rho (Lane-1), only mCherry (Lane-2), and Rho-mCherry fusion protein (Lane-3). Blots probed with anti-mCherry antibody detected prominent bands of free mCherry and Rho-mCherry in lanes 2 and 3, respectively. Lane-1 showed no other band except a non-specific band, which was detected in all the samples. (C) Growth analysis of *E. coli* MG1655 expressing only Rho and Rho-mCherry fusion protein showed similar profiles, indicating that mCherry fusion with Rho does not affect Rho activity. The growth curves represent average values obtained from three individual colonies with SEM as error bars.

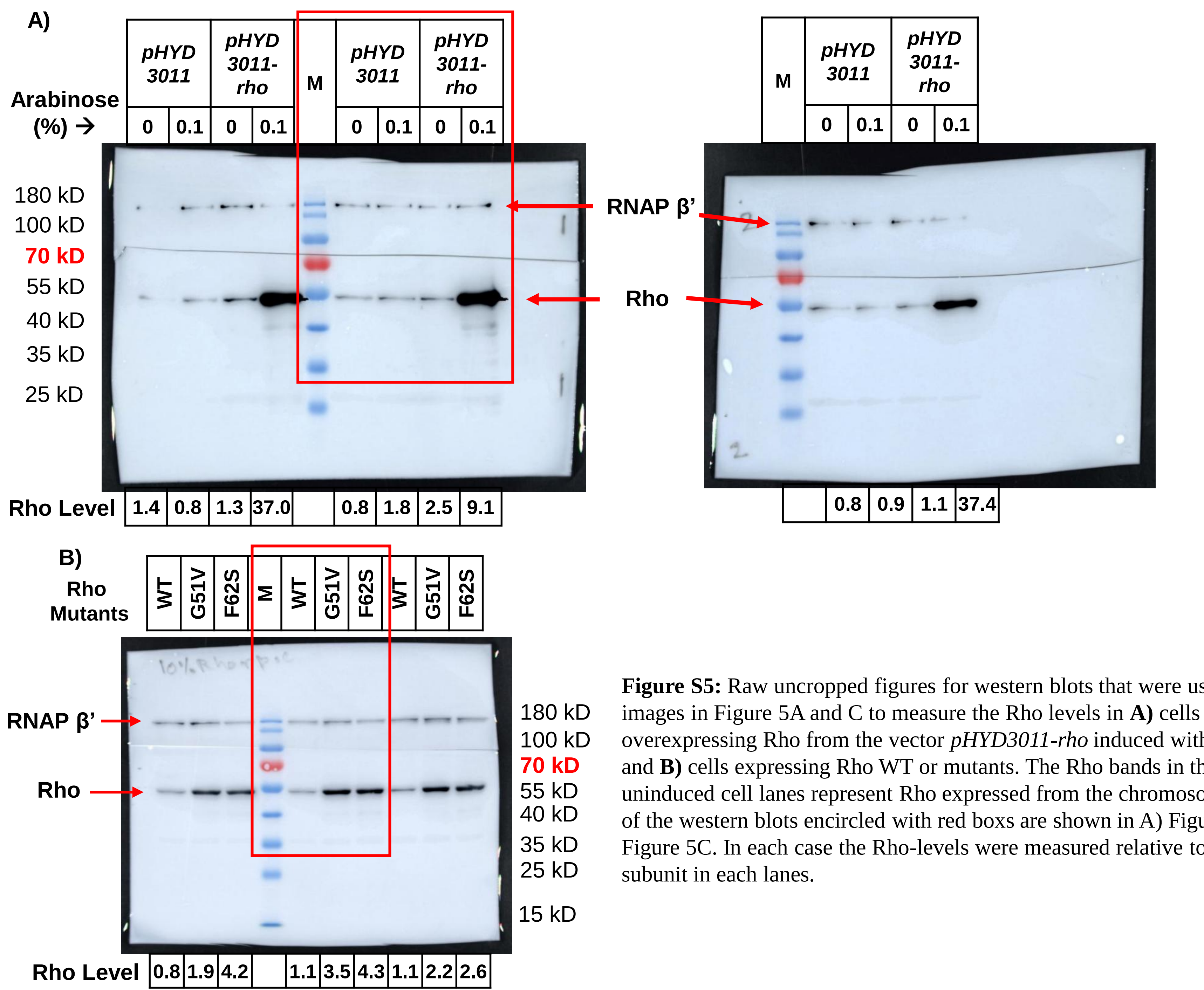

Figure S5: Raw uncropped figures for western blots that were used to prepare the images in Figure 5A and C to measure the Rho levels in **A)** cells overexpressing Rho from the vector *pHYD3011-rho* induced with 0.1 % Arabinose and **B)** cells expressing Rho WT or mutants. The Rho bands in the empty vector or uninduced cell lanes represent Rho expressed from the chromosomal rho. The part of the western blots encircled with red boxs are shown in A) Figure 5A and B) Figure 5C. In each case the Rho-levels were measured relative to RNAP β' subunit in each lanes.

Table S1: Average dissociation constants (K_d) for Rho-DNA interactions

A) K_d values for Rho WT-different DNAs interactions

**DNA (kB)**	**Av, K_d (nM)**
0.6 kb (pRS22)	Could not fit
1.3	191.7
1.5	144.6
2.0	74.3
3.5 at 37°C	25.9
3.5 at 4°C	12

B) K_d values of WT and Mutant Rho-3.5 kB DNA interactions

**Rho Mutant**	**Av. K_d (nM)**
WT	25.9
G51V	29.9
F62S	86.5
Y80C	180.0

Table S2: Plasmids and oligos used in this study

**Plasmids**:	**Description**	**References**
pRS22	Plasmid vector pTL61T carrying P_{T7A1}-*nutR-TR'-T1T2-LacZ* sequence (Amp^R)	[1]
pRS96	*E. coli rho*-his cloned at XhoI/NdeI sites of pET21b (Amp^R)	[6]
pRS106	Plasmid pK8641 carrying P_{T7A}*1-trpt'-lacZAY* sequence (Amp^R)	[2]
pRS317	Rho WT cloned in pCL1920 under *rho* promoter ($Spec^R$)	[3]
pRS632	*E. coli rho* cloned in pHYD3011 at NdeI/Sal I sites (Amp^R)	
pRS669	pHYD3011, Modified pBAD18 vector with the MCS and RBS of pET vector, (Amp^R)	[4]
pRS1784	*E. coli rho* Y80C cloned at XhoI / NdeI sites of pET21b (Amp^R)	[6]
pRS2246	Only *mCherry* cloned in pCL1920 under *rho* promoter ($Spec^R$)	This study
pRS2264	*mCherry* fused at the N-terminal of *rho* with linker $(GGGGS)_3$ in pCL1920 under *rho* promoter ($Spec^R$)	This study
pRS1246	Rho F62S in pCL1920 generated by SDM of pRS317 ($Spec^R$)	[5]
pRS1113	Rho G51V in pCL1920 generated by SDM of pRS317 ($Spec^R$)	[6]
pRS378	Rho G51V - pET21b, C-terminal His-tagged (Amp^R)	[6]
pRS2352	Rho F62S - pET21b, C-terminal His-tagged (Amp^R)	This study
**Oligos:**		
RSRK-1	GTTTTCCCAGTCACGAC	
RK23B	TGGAGTTCCAGACGATACG	
RS58	ATAAACTGCCAGGAATTGGGGATC	
RS83	5'- Biotinylated RS58	
RS177	GAATTGTGAGCGCTCACAATTCGGATATATATTAACAATTACCTG	
RS1743	CAGATCGGTCATCAATGCGT	
RS1744	AACAGAAACAGTGTCGTGAA	
RS2086	ATAACGCCGACTTCGATGGT	
RS2087	GATGATTGGTTCGCCGTTCG	
RS2213	GCACCCCAGGCTTTACACTTT	
RS2214	ATGTGCTGCAAGGCGATTAAG	
RS2243	GGAGGAGGAGGATCAGGAGGAGGAGGATCAGGAGGAGGAGGAT CAATGAATCTTACCGAATTAAAGAAT	
RS2303	CTCCTCGCCCTTGCTCACCATAGTGGTGTGAGTTCTTAAACTTG	
RS2304	AGTTTAAGAACTCACACCACTATGGTGAGCAAGGGCGAGGAG	
RS2309	TGATCCTCCTCCTCCTGATCCTCCTCCTCCTGATCCTCCTCCTCCCT TGTACAGCTCGTCCATGCCGCCGGT	
RS2329	GCGCGCGAGCTCCCTCGACGCTAACCTGGCGTAAGGGAATTTG	
RS2461	AATCTCTATCGTGCGGTGG	
RS2462	TGCACCATCGTCTGCTCATCC	

References:

1 Cheeran, A., Babu Suganthan, R., Swapna, G., Bandey, I., Achary, M. S., Nagarajaram, H. A., et al. (2005) Escherichia coli RNA polymerase mutations located near the upstream edge

of an RNA: DNA hybrid and the beginning of the RNA-exit channel are defective for transcription antitermination by the N protein from lambdoid phage H-19B. *J Mol Biol*. **352**, 28-43, https://doi.org/10.1016/j.jmb.2005.06.052.

2 Pani, B., Banerjee, S., Chalissery, J., Muralimohan, A., Loganathan, R. M., Suganthan, R. B., et al. (2006) Mechanism of inhibition of Rho-dependent transcription termination by bacteriophage P4 protein Psu. *J Biol Chem*. **281**, 26491-26500, https://doi.org/10.1074/jbc.M603982200.

3 Harinarayanan, R. and Gowrishankar, J. (2003) Host factor titration by chromosomal R-loops as a mechanism for runaway plasmid replication in transcription termination-defective mutants of Escherichia coli. *J Mol Biol*. **332**, 31-46, https://doi.org/10.1016/s0022-2836(03)00753-8.

4 Pani, B., Ranjan, A. and Sen, R. (2009) Interaction surface of bacteriophage P4 protein Psu required for complex formation with the transcription terminator Rho. *J Mol Biol*. **389**, 647-660, https://doi.org/10.1016/j.jmb.2009.04.052.

5 Shashni, R., Qayyum, M. Z., Vishalini, V., Dey, D. and Sen, R. (2014) Redundancy of primary RNA-binding functions of the bacterial transcription terminator Rho. *Nucleic Acids Res*. **42**, 9677-9690, https://doi.org/10.1093/nar/gku690.

6 Chalissery, J., Banerjee, S., Bandey, I. and Sen, R. (2007) Transcription termination defective mutants of Rho: role of different functions of Rho in releasing RNA from the elongation complex. *J Mol Biol*. **371**, 855-872, https://doi.org/10.1016/j.jmb.2007.06.013.

Table S3: Raw data for the RT-qPCR (figure 5B) and the β- galactosidase activity (figure 5D)

A) Raw data for fold change in *lacZ* expression as obtained from the RT-qPCR

		Set-1	Set-2	Set-3	**Avg**	**SD**	t-test p-Values	Symbol
pHYD3011	Uninduced	1.00	1.00	1.00	**1.00**	**0.00**		
	Induced	0.43	0.51	0.62	**0.52**	**0.08**	0.0010	**
pHYD3011-Rho	Uninduced	1.00	1.00	1.00	**1.00**	**0.00**		
	Induced	0.16	0.13	0.34	**0.21**	**0.09**	0.0002	***

The statistical significance of the results was assessed by the unpaired two-tailed Student's t-test using Microsoft Excel 2016 by comparing the fold change in *lacZ* expression levels in induced culture with that for uninduced culture in each group with p-values indicated by * $p< 0.05$, ** $p<0.01$, *** $p<0.001$, **** $p<0.0001$

B) Raw data for the β- galactosidase activity (Miller units) in different sets of *E. coli* strain RS1993*Δrho* expressing *rho* WT or mutants from the vector pCL1920

**Rho**	Set-1	Set-2	Set-3	Set-4	**Average**	**SD**	t-test p-Values	Symbol
**WT**	2726.04	2840.23	3331.32	3226.08	**3030.92**	**253.79**		
**G51V**	2238.25	2697.54	2279.86	2202.19	**2354.46**	**199.97**	0.011	*
**F62S**	2225.11	2226.05	1566.99	2251.64	**2067.45**	**289.13**	0.005	**

The statistical significance of the assays was assessed by the unpaired two-tailed Student's t-test using Microsoft Excel 2016 by comparing the values of the β- galactosidase activity for mutant strains with that for WT strain with p-values indicated by * $p< 0.05$, ** $p<0.01$, *** $p<0.001$, **** $p<0.0001$
